# Cognitive Dysfunction in Multiple Sclerosis: Educational Level as a Protective Factor

**DOI:** 10.3390/neurolint13030034

**Published:** 2021-07-22

**Authors:** Mar Estrada-López, Sheila García-Martín, Isabel Cantón-Mayo

**Affiliations:** 1 Department of Psychology, Sociology and Philosophy, Faculty of Education, University of León, 24071 León, Spain; mestl@unileon.es; 2 Department of General and Specific Didactics and Educational Theory, Faculty of Education, University of León, 24071 León, Spain; icanm@unileon.es

**Keywords:** cognitive dysfunction, educational level, multiple sclerosis, protective factor, RRMS, SPMS

## Abstract

Most people with MS experience cognitive deficits especially in attention, memory, information processing, and executive functions, negatively impacting on their quality of life. Cognitive variables of short-term memory, logical memory, and verbal fluency in 65 patients with multiple sclerosis (MS) were analysed in conjunction with sociodemographic variables such as sex, age, and educational level that might influence disease progression. We found that psychoeducational variables exerted a significant effect on the cognitive status of patients with MS. Thus, when considering sex, age, educational level, and type of MS (SPMS or RRMS), tests for between-subject effects revealed statistically significant differences in all three cognitive variables. In addition, we found that the type of MS and time since onset also generated significant cognitive differences. Our study shows that educational achievement or level is a protective factor against the disease, acting as a source of intellectual enrichment that promotes cognitive reserve in patients with MS. Further longitudinal studies assessing disease progression and prognosis in patients with MS would be useful in order to determine the specific importance of these variables in such patients and in strategies that could enhance their performance in neuropsychological assessment tasks.

## 1. Introduction

Multiple sclerosis (MS) is a chronic inflammatory disorder of the central nervous system that attacks myelinated axons and causes damage to grey matter and/or loss of cellular connectivity due to injury and degeneration of white matter [1,2,3].

Almost 80% of people with MS experience cognitive deficits, especially in attention, memory, information processing, and executive functions [4], negatively impacting on their quality of life [5,6].

Among the sub-types of the disease, this study focused on two sub-types: relapsing remitting multiple sclerosis (RRMS) and secondary progressive multiple sclerosis (SPMS). The initial form of MS is often RRMS, which usually begins with visual, sensory, and/or motor symptoms and is frequently associated with few attacks and subsequent partial or complete recovery [7].

Memory dysfunction is one of the first signs of MS [8], and studies have reported effects on memory and processing speed [4,8]. Short-term memory refers to functions associated with daily living such as remembering names, faces, addresses, locations of objects, future events and appointments, and research indicates that it is one of the functions most highly affected by the disease, negatively affecting the everyday activities of people with MS [4].

Studies have shown that verbal fluency is also affected in 40–64% of patients with MS [9]. Verbal fluency is frequently used in neuropsychological assessments because the evidence indicates that it is a sensitive test for diagnosing cognitive impairment in MS [10].

Previous studies have also reported significant correlations in people with MS between their performance in visuospatial neuropsychological tasks and their cognitive status, physical disability, and neurological signs affecting the pyramidal tract, cerebellum, and brainstem [11,12]. However, no significant correlation has been reported between a deficit in visuospatial abilities and other neuropsychological variables such as time since onset, type of MS, depression, or medication status.

Relatively recent research has shown that other variables such as intellectual enrichment can attenuate cognitive decline in patients with MS [13], using information processing speed as a measure to collect data. The speed with which information is processed has been identified as the cognitive capacity most severely affected by MS and is therefore representative of cognitive decline [14].

The aim of our study was to analyse short-term memory, logical memory, and verbal fluency in 65 people with MS in conjunction with other sociodemographic variables that might influence disease progression. These variables were age, educational level, sex, and type of MS. Our goal was to determine the specific importance of these variables in these patients and in strategies that could enhance their performance in neuropsychological assessment tasks and attenuate their cognitive decline.

## 2. Materials and Methods

The study sample consisted of 65 people diagnosed with MS. The diagnosis was confirmed by a neurologist using the McDonald criteria [15,16]. Of the 65 patients, 49 had RRMS and 16 had SPMS. Participants were randomly recruited from the Multiple Sclerosis patient population in León (city and province), Spain. Recruitment and the two evaluations required for this study were completed by neurologists at the University Hospital of León.

This was a descriptive cross-sectional study. All subjects gave their written informed consent prior to participation in the study, which was conducted in accordance with the Declaration of Helsinki and approved by the scientific and ethics committees.

Sociodemographic data for the study sample were collected during an interview. Participants’ memory and verbal fluency were assessed using, respectively, the Luria-DNA battery and the primary mental abilities battery. The Luria-DNA battery [17] is a highly respected neuropsychological assessment instrument for the assessment of cognitive decline. This battery comprises 5 subtests that each test a range of memory functions and abilities in key areas of cognition. In the present study, we administered the two subtests that assess the area of memory, specifically, short-term memory and logical memory. The primary mental abilities battery was developed by Thurstone et al. between 1936 and 1968 [18]. This battery consists of five subtests that assess specific mental abilities: verbal, spatial, logical reasoning, and numerical and verbal fluency. For this study, we used only the verbal fluency test, which assesses the subject’s speaking and writing abilities as well as testing their vocabulary level. Verbal ability is related to inhibition and other executive processes, such as working memory and attention. Thus, the particular tasks on the verbal fluency test include giving subjects a particular time period during which they must list as many words as they can think of that begin with a specified letter [19].

## 3. Results

### 3.1. Descriptive Analyses

We performed descriptive analyses of the sociodemographic variables. The study sample (*n* = 65) contained 49 subjects with RRMS and 16 with SPMS.

Of those with RRMS (see Table 1), 83.7% were women (*n* = 41), 16.3% were men (*n* = 8), and 61.2% were aged below 40 years old. By educational level, primary education (6 to 8 years of formal education) accounted for 30.6% of the sample, secondary education (8 to 12 years of formal education) accounted for 42.9%, and higher education (15 years or more of formal education) accounted for 26.5%; meanwhile, 55.1% reported living with other(s) and 95.9% were not retired.

Of the 16 people with SPMS (see Table 2), 56.2% were men (*n* = 9) and 43.8% were women (*n* = 7). Most (68.7%) were aged 40 years old or more. By educational level, primary education (6 to 8 years of formal education) accounted for 12.5% of the sample, secondary education (8 to 12 years of formal education) accounted for 56.2%, and higher education (15 years or more of formal education) accounted for 31.3%. The majority (68.7%) lived with other family members, while the remaining 31.3% lived alone. Only 31% were retired.

### 3.2. Multivariate Linear Analysis

Parametric tests are generally preferred over non-parametric tests as they are more powerful in terms of their ability to detect significant relationships between variables. This type of test also works well with non-normally distributed data where the sample size is sufficiently large and give reliable results even for groups with differing amounts of variability. Given the sample size (*n* = 65), we needed to ensure that the distribution of sample means approached a normal distribution (central limit theorem). This proved to be the case, and thus, our primary analysis involved parametric tests.

The first step in our analysis was the use of multivariate linear analysis to determine the relationships between the psychoeducational variables of sex, age, educational level, and type of MS, and those of variables of logical memory, short-term memory, and verbal fluency.

When considering sex, age, educational level, and type of MS, tests for between-subject effects showed statistically significant differences in logical memory, short-term memory, and verbal fluency. In relation to *sex*, we found statistically significant differences when comparing short-term memory between men and women with MS, whereby this cognitive process was less severely affected in women (W_women_ = 35.74 vs. M_men_ = 25.26; *p* < 0.005).

However, we did not observe any statistically significant differences between groups for any of the cognitive variables analysed when *age* was used as the grouping variable. Nevertheless, a scatter graph confirmed an inverse linear relationship between *age* and verbal fluency, whereby verbal fluency decreased as age increased.

Participants were divided into three *educational level* groups: *primary education*, comprising those who had only completed basic general education or compulsory secondary education that involved 6 to 8 years of formal education; *secondary education* for those who had only competed vocational training, a university entrance course, and/or non-compulsory secondary education that involved 8 to 12 years of formal education; and *higher education* for those who had completed a university diploma course, an undergraduate degree, and/or a doctorate that entailed 15 years or more of formal education. Multivariate analyses revealed statistically significant differences in verbal fluency when considering *educational level* (E_higher_= vs. E_primary_=; *p* < 0.005), whereby verbal capacity was best conserved in participants with a higher educational level (see Table 3). Participants with a primary education obtained a mean score of 21.03%, whereas those with a higher education obtained a mean score of 36.44%.

In relation to *type of MS*, no statistically significant differences were observed between this and logical memory, short-term memory, or verbal fluency. However, we did find a negative correlation between variables whereby SPMS correlated with a lower score for logical memory, short-term memory, and verbal fluency. Hence, the more severe the disease, the worse the scores obtained for these variables (see Table 4).

Lastly, when we compared the three cognitive variables, we observed significant correlations between the two types of memory studied and verbal fluency (M_short-term_ = 48.69 and F_verbal_ = 31.28; *p* < 0.001), (M_logic_ = 50.62 and F_verbal_ = 31.28; *p* < 0.027). The same was not the case between the types of memory analysed (see Table 5). These results may be due to the type—phonological—of verbal fluency test employed, since the task required participants to inhibit words that did not begin with a specified letter and implement strategies to generate the highest possible number of words within the stipulated time. Moreover, besides serving as an indicator of the subject’s level of vocabulary, this ability is also closely related to inhibition and other executive processes, such as working memory and attention.

### 3.3. Non-Parametric Tests

Non-parametric analysis (Kruskal–Wallis test) was used to determine whether educational level affected the following variables: logical memory, short-term memory, verbal fluency, and level of disability (EDSS). The results are presented in Table 6.

This analysis demonstrates that higher educational levels were associated with higher scores in logical memory and verbal fluency tests.

### 3.4. Multivariate Logistic Regression

Multivariate logistic regression was performed to assess the relationship between level of formal education and the variables of short-term memory, logical memory, verbal fluency, and level of disability. Results for the model (x92846 = 196,460 (*p* < 0.001)) are shown in Table 7.

Statistically significant differences were found for all the variables studied except that of short-term memory.

## 4. Discussion

The overall objective of the present study was to examine the level of cognitive dysfunction in the variables of short-term memory, logical memory, and verbal fluency in subjects with MS. Previous studies [20] have found that educational level correlates positively with the total production of words remembered. We also found that patients with a higher educational level maintained a better verbal capacity, obtaining higher scores than those with a primary education.

Hence, in our study, a high educational level was associated with better conservation of verbal capacity in patients with MS. This finding agrees with the results reported in other studies [21,22], which found a positive correlation between the phonological verbal fluency task and educational level.

In addition, we confirmed that the type and time since onset of MS generated significant cognitive differences, whereby the verbal fluency of patients with SPMS was more severely affected than that of patients with RRMS, again confirming the findings reported in previous studies [23].

Meanwhile, the origins of memory disorder in MS remain a subject of debate. Classical theory attributes this dysfunction to deficits in retrieval processes [24], but more recent studies have reported evidence to support the hypothesis that it is due to alterations in coding processes [25,26]. In addition, several studies have linked cardiovascular comorbidities to poor motor progression of MS [11,12,13,14] and cardiovascular risk factors have also been shown to have a negative impact on neuropsychological performance and, more generally, on brain integrity, although a causal relationship has not yet been determined [27]. Thus, it is possible to say that there are numerous coexisting and interacting factors working together to produce the various different effects on cognitive performance seen in MS patients.

The progressive nature of cognitive dysfunction is a well-known feature of MS-related disability; differences in sample size by sex is frequently observed in MS, since women present a higher prevalence [28,29]. However, our study shows that educational level is a potentially protective factor against the disease, acting as a source of intellectual enrichment that provides a cognitive reserve for MS patients, so helping to mitigate cognitive decline [30].

This study has some methodological limitations, in particular its small sample size and cross-sectional design. In addition, participant interviews did not include a question concerning the duration of their disease.

Considering these limitations in light of our results, perhaps the most important area to address is the study design. Longitudinal studies of MS are rare, and due to the promising results concerning educational level and cognitive decline, we feel it would be useful to carry out a long-term longitudinal study in which newly diagnosed participants were grouped according to educational level and time since onset in order to determine whether these associations and correlations remain significant as regards long-term prognosis and this a putative protective effect.

In view of our results and given the scarcity of research that is not cross-sectional, it would be useful if longitudinal studies were conducted to assess disease progression and prognosis in patients with MS and analyse predictors of cognitive decline in order to determine probable progression. In addition, a new area of research we would like to consider is the use of Magnetic Resonance Imaging (MRI) to track changes in the brain structure of MS patients’ progression of MS. The use of this tool would be an invaluable additional data source in any future longitudinal study of MS. For instance, a study published since the first draft of this manuscript [31] used MRI to correlate cognitive dysfunction in MS sufferers with structural, functional, and metabolic changes in the brain. It is possible that greater knowledge, particularly concerning changes in brain metabolism, might assist in attempts to delay the impact of cognitive and physical decline in people with MS and perhaps endow them with effective tools to better cope with the disease itself following their diagnosis.

## Figures and Tables

**Table 1:** Sociodemographic data for patients with RRMS.

Age	20–40	40 or over		
	30 (61.2%)	19 (38. 8%)		
Educational level	Primary	Secondary	Higher	
	15 (30.6%)	21 (42.9%)	13 (26.5%)	
Living circumstances	Living alone	Living with other(s)		
	22 (44.9%)	27 (55.1%)		
Retired	Yes	No		
	2 (4.1%)	47 (95.9%)		*n* = 49

**Table 2:** Sociodemographic data for patients with SPMS.

Age	20–40	40 or over		
	5 (31.3%)	11 (68.7%)		
Educational level	Primary	Secondary	Higher	
	2 (12.5%)	9 (56.2%)	5 (31.3%)	
Living circumstances	Living alone	Living with other(s)		
	5 (31.3%)	11 (68.7%)		
Retired	Yes	No		
	5 (31.3%)	11 (68.7%)		*n* = 16

**Table 3:** Relationships between cognitive variables and educational level in the multiple sclerosis sample.

Cognitive Variables	Primary Education (*n* = 17)	Secondary Education (*n* = 30)	Higher Education (*n* = 18)	
	M	M	M	*p*-Value
Short-term memory	30.29 ± 10.77	50.83 ± 11.52	46.39 ± 14.07	0.796
Logical memory	29.71 ± 21.48	51.83 ± 15.72	52.50 ± 14.01	0.307
Verbal fluency	21.03 ± 11.48	33.50 ± 10.83	36.44 ± 17.64	0.002
EDSS	2.58 ± 1.41	3.00 ± 1.87	2.44 ± 1.19	0.462

EDSS: Extended Disability Status Scale.

**Table 4:** Relationships between EDSS, educational level, cognitive variables, and type of MS.

Variables	RRMS	SPMS	*p*
	(*n* = 49)	(*n* = 16)	
EDSS			
No Disability	27 (55.1%)	2 (12.5%)	
Minimum to Moderate	19 (38.8%)	6 (37.5%)	<0.001
Affecting Walking	3 (6.1%)	8 (50%)	
EDUCATION LEVEL			
Primary Education	15 (30.6%)	2 (12.5%)	0.368
Secondary Education	21 (42.9%)	9 (56.2%)	
Higher Education	13 (26.5%)	5 (31.3%)	
COGNITIVE VARIABLES			
Logical Memory (M ± SD)	51.33 ± 17.04	48.44 ± 17.76	0.562
Short-Term Memory (M ± SD)	50.71 ± 10.80	42.50 ± 13.90	0.017
Verbal Fluency (M ± SD)	32.22 ± 14.02	28.38 ± 14.87	0.351

EDSS: Extended Disability Status Scale; M ± SD: Average (arithmetical mean) plus or minus standard deviation.

**Table 5:** Relationships between cognitive variables.

Cognitive Variables	Mean	Logical Memory vs. Short-Term Memory	Logical Memory vs. Verbal Fluency	Short-Term Memory vs. Verbal Fluency
	M	*p*	*p*	*p*
Logical Memory	50.62	0.054	0.027	-
Short-Term Memory	48.69	0.054	-	0.000
Verbal Fluency	31.28	-	0.027	0.000

**Table 6:** Relationships between cognitive variables and educational level.

Cognitive Variables	Primary Education (*n* = 17)	Secondary Education (*n* = 30)	Higher Education (*n* = 18)	
	M	M	M	*p*
Short-Term Memory	30.29	36.42	29.86	0.396
Logical Memory	29.71	33.87	34.67	0.725
Verbal Fluency	21.03	36.20	38.97	0.009
EDSS	31.88	35.52	29.86	0.573

**Table 7:** Multivariate regression results.

Short-Term Memory	Logical Memory	Verbal Fluency	EDSS	
27696	17052	33178	23850	X^2^
0.067	0.048	0.000	0.005	*p*
